# A Pipeline for Classifying Deleterious Coding Mutations in Agricultural Plants

**DOI:** 10.3389/fpls.2018.01734

**Published:** 2018-11-28

**Authors:** Maxim S. Kovalev, Anna A. Igolkina, Maria G. Samsonova, Sergey V. Nuzhdin

**Affiliations:** ^1^Department of Applied Mathematics, Peter the Great St.Petersburg Polytechnic University, St. Petersburg, Russia; ^2^Program Molecular & Computational Biology, Dornsife College of Letters Arts and Science, University of Southern California, Los Angeles, CA, United States

**Keywords:** deleterious mutation, random forest (bagging) and machine learning, *Orýza*, *Pisum*, *Cicer*

## Abstract

The impact of deleterious variation on both plant fitness and crop productivity is not completely understood and is a hot topic of debates. The deleterious mutations in plants have been solely predicted using sequence conservation methods rather than function-based classifiers due to lack of well-annotated mutational datasets in these organisms. Here, we developed a machine learning classifier based on a dataset of deleterious and neutral mutations in *Arabidopsis thaliana* by extracting 18 informative features that discriminate deleterious mutations from neutral, including 9 novel features not used in previous studies. We examined linear SVM, Gaussian SVM, and Random Forest classifiers, with the latter performing best. Random Forest classifiers exhibited a markedly higher accuracy than the popular PolyPhen-2 tool in the *Arabidopsis* dataset. Additionally, we tested whether the Random Forest, trained on the *Arabidopsis* dataset, accurately predicts deleterious mutations in *Orýza sativa* and *Pisum sativum* and observed satisfactory levels of performance accuracy (87% and 93%, respectively) higher than obtained by the PolyPhen-2. Application of Transfer learning in classifiers did not improve their performance. To additionally test the performance of the Random Forest classifier across different angiosperm species, we applied it to annotate deleterious mutations in *Cicer arietinum* and validated them using population frequency data. Overall, we devised a classifier with the potential to improve the annotation of putative functional mutations in QTL and GWAS hit regions, as well as for the evolutionary analysis of proliferation of deleterious mutations during plant domestication; thus optimizing breeding improvement and development of new cultivars.

## Introduction

New mutations continuously arise in populations. Some of them are neutral, but many are deleterious (Grossman et al., 2010). Under most circumstances, natural selection is effective in maintaining strong deleterious mutations at low level, however mildly deleterious variants may reach considerable frequency in populations due to hitchhiking and population bottlenecks. Deleterious variants may affect phenotypic traits and decrease organismal fitness. Quite the opposite, in maize intermediate and weakly deleterious alleles are involved in heterosis (Yang et al., 2017). In human rare, deleterious SNPs are associated with common diseases and cancer (Taylor et al., 2015). Therefore, it is no wonder that estimation of the deleterious mutations prevalence in different species is a topic of vivid interests.

Theoretical predictions place the fraction of deleterious mutations in barley, soybean, rice, maize, and *Arabidopsis* genomes from 20% to 40% approximately (Günther and Schmid, 2010; Mezmouk and Ross-Ibarra, 2014; Kono et al., 2016). Deleterious alleles are usually at low frequency, an observation that is in agreement with the action of weak purifying selection. The prevalence of deleterious alleles differs between wild species, landraces, and elite cultivars. Using rice sequences Günther and Schmid (2010) found fewer deleterious substitutions in the wild than in cultivated rice. In comparisons with traditional landraces, elite maize inbreds show an increase in the proportion of deleterious variants fixed within the population, but the much smaller proportion of segregating deleterious variants (Yang et al., 2017). This is explained by bottlenecks during modern breeding that results in fixation of the majority of mutations, therefore reducing a fraction of segregating variation.

The issue of deleterious variation in plant genotypes is particularly essential for crop improvement, because crop productivity may be reduced due to a persistence of deleterious variants at a moderate frequency. Indeed Yang et al. (2017) found that deleterious variants may contribute substantially to variation in fitness-related quantitative traits in maize and that incorporation of information about deleterious mutations may improve existing genomic prediction frameworks.

NGS technologies open a way to annotate the functional effect of individual SNPs. As the regulatory code responsible for gene activity still remains a puzzle, only genetic variants in the coding regions are considered. The general belief is that non-synonymous substitutions may change protein structure and therefore many of them should have the deleterious effect on protein function, which in turn manifests as biochemical or morphological mutations. The methods for prediction of deleterious effects of non-synonymous substitutions in proteins could be subdivided into two groups. The first group methods exploit sequence conservation and are based on the assumptions that SNPs in evolutionarily conserved regions are likely to be deleterious. Some of them like SIFT use simple cut-off to discriminate deleterious variants from neutral (Sim et al., 2012), while other like MAPP (Stone and Sidow, 2005) and GERP+++ (Davydov et al., 2010) employ phylogenetic information in addition.

The machine learning algorithms lay the foundation of the second group methods. Of these the most widely used is PolyPhen-2 (Adzhubei et al., 2010). This method employs the rigorously annotated datasets of human disease-causing mutations for training that preconditions its high predictive accuracy. As a machine learning method PolyPhen-2 consists of three steps: firstly a set of features that characterize a mutation was extracted using sequence characteristics, multiple alignment scores, and information about the 3D structure of the resulting protein. At the next steps, training and cross-validation were performed followed by classification with a naïve Bayes approach. It should be noted, that being trained on human data, PolyPhen-2 is sometimes applied to predict deleterious mutations in other species. There is, however, little consensus about the eligibility of such a direct knowledge transfer. Indeed, it is known that alleles annotated as deleterious in humans at about 15% of cases correspond to normal alleles in other mammals (Kondrashov et al., 2002). It appears from this that to achieve more accurate predictions training might have to be separately executed species by species. However, for many species, information required for classifier training might be substantially more limited than for humans. Accordingly, the question arises whether it is possible to use the information obtained for one species for the search for harmful mutations in another, perhaps phylogenetically close, species.

This question has long been discussed in machine learning in the following formulation: how to transfer knowledge from one object to another, considered to be close (in the sense of data sampling distribution), to solve a specific problem (whether classification or regression). A set of methods that provide the methodology for solving such problems is denoted Transfer Learning (TL). These methods have found broad application in many practical problems. For instance, Lagunas and Garces (2017) classify the painted images of various objects using their naturalistic form (photos). Closer to home, Transfer Learning was used for evaluating the quality of protein models (Hurtado et al., 2018), the localization of proteins in the cell based on ontology databases (Mei et al., 2011) and the search for associations between the genome and the phenotype (Petegrosso et al., 2018).

Up to now, most publications predicting deleterious mutations in plants use sequence conservation methods that is mostly due to lack of well-annotated datasets of deleterious and neutral mutations in these organisms. However, recently, Kono et al. (2016) have assembled a validated database of 2,910 function-altering mutations in *Arabidopsis* that opens the way for development of machine learning methods specifically tailored for plants. Here, we developed the Random Forest classifier that being tested on two plant species – *Orýza sativa* and *Pisum sativum* – for which the sufficient number of neutral and functional mutations are known – showed substantially better performance than PolyPhen-2. We also attempted to improve our classifier using the approaches of Transfer learning, as this technique could provide knowledge transfer from one species for which a lot of information is available to a close species with limited information. Finally, we validate this classifier using population data on single nucleotide allele frequency available for *Cicer arietinum* (Plekhanova et al., 2017). We believe our classifier will be helpful in plant research for prioritizing mutations in QTL and GWAS support intervals for functional validation, for developing GRN-based models to solve the genotype-to-phenotype problem, as well as for improvement of breeding programs.

## Materials and Methods

### *Arabidopsis* Training Database

The list of amino acid substitutions in *Arabidopsis thaliana* proteins was obtained from the database created by Kono et al. (2018). The database consists of 13,707 replacements available, of them 4,409 were labeled mutations in 994 proteins: 2,894 deleterious and 1,515 neutral. The protein sequences were downloaded from “The *Arabidopsis* Information Resource.”

### *Orýza sativa* and *Pisum sativum* Test Datasets

The sets of deleterious mutations in rice (*O. sativa*) and pea (*P. sativum*) were extracted from the UniProt mutation database (The UniProt Consortium, 2017). To construct a set of neutral mutations in rice and pea BLASTp program (Altschul et al., 1997) was used to align each protein sequence against SwissProt sequence database (Bairoch, 1996) and proteins with more than 95% identity to a query sequence were selected. At the next step, the selected sequences were multiply aligned with Clustal Omega (Sievers and Higgins, 2014) and a set of neutral mutations was generated under the following rule. We consider amino acid substitutions without any known phenotype, not present in a continuous block of substituted residues (i.e., are isolated) and independent (i.e., there were no other substitutions in the same sequences of alignment). This rule makes it possible to avoid the phenomenon of correlated mutational behavior between columns in multiple sequence alignment (Kowarsch et al., 2010). Besides we consider only alignment columns that have no more than one substitution. To balance the datasets, neutral mutations were randomly downsampled so that their number was equal to the number of deleterious mutations. Overall, the dataset for rice contained 764 mutations in 400 proteins (by 382 deleterious and neutral); the pea dataset contained 136 mutations in 60 proteins (by 68 deleterious and neutral).

### *Cicer arietinum* Target Dataset

433 *Cicer arietimum* landraces from N. I. Vavilov All-Russian Institute for Genetic Resources (VIR collection) were genotyped by GBS sequencing and variants were called and filtered following standard criteria; overall 56855 SNPs were identified (Plekhanova et al., 2017). Identification of SNPs in protein coding regions and classification of those into synonymous and non-synonymous classes was done with SnpEff tools (Cingolani et al., 2012): 3023 synonymous and 3467 non-synonymous replacements were determines within 2569 proteins.

### Classifier Features

The set of classification features was aggregated by different methods. To extract a set of features characterizing substitutions, the PolyPhen-2 web service (Adzhubei et al., 2010) was used. Additional servers and sources of information were also involved, such as the PfamScan (Finn et al., 2014) and the PCI-SS (Green et al., 2009). The former was used to check whether the amino acid substitution locates within a protein domain of the Pfam database. Features obtained with the latter service incorporate information about the secondary structure of the protein in the loci of the substitution. Since information about the three-dimensional structure of a target protein is not always known, these features played the role of alternative structural characteristics. PCI-SS server indicates a protein secondary structure – α-helix, β-sheet, or non-regular structure – which contains the substitution of interest, and also provides three quantitative characteristics about the structural state of the target amino acid in the protein based on the mean-square error between the models considered in the PCI-SS algorithms. To evaluate the physicochemical nature of amino acid substitutions, several measures were used: the Grantham distance (Grantham, 1974), the Sneath index (Sneath, 1966), the Epstein’s coefficient of difference (Epstein, 1967), and the Miyata distance (Miyata et al., 1979). The quantitative evaluation of the amino acid substitution by the matrix of BLOSUM62 substitutions was added as an extra feature (Henikoff and Henikoff, 1992).

Two additional features have been constructed that take into account the amino acid context around the mutation position. The first feature was defined as the mean distance over the Grantham matrix between the wild-type amino acid in the mutation position and each of the two neighboring amino acids. The second feature was calculated in the same way but considering two amino acids from a mutant position at a distance of one. The construction of these features was based on the following hypothesis: if the amino acids that are very different in their physicochemical properties are next to each other, this is most likely justified by the constraints on functions to be performed. Therefore, the more physicochemical differences are in the amino acid position from its context, the more likely it is for the mutation in the position of this amino acid to be harmful.

### Classifiers

To solve the classification problem of mutations to deleterious versus neutral, three classifiers were tested: Support Vector Machines with a linear kernel (Linear SVM), Support Vector Machines with a Gaussian kernel (Gaussian SVM) (Cristianini and Shawe-Taylor, 2000), and Random Forest (RF) (Breiman, 2001). The Linear SVM method is based on the search for a separating hyperplane with the maximum gap between the data. To use a non-linear separation of classes, the Gaussian SVM was examined; it utilizes the Gaussian kernel instead of the scalar product in the Linear SVM (Cristianini and Shawe-Taylor, 2000). The RF uses the ideas of bagging, or Bootstrap Aggregating (a composition of independent classifiers, in this case, of decision trees) and the method of random subspaces (description of objects using subspaces of the feature space) (Breiman, 2001).

The choice of hyperparameter values for classifiers was carried out on the *Arabidopsis* dataset. For each classifier, the traditional procedure – grid search with fivefold cross-validation – was performed to find the optimal values of hyperparameters. These values are usually selected as the values that provide the highest cross-validation score that leads to the preventing of overfitting. Further, the optimal hyperparameters were utilized while classifiers’ training. One might see that the overfitting effect was not observed (Supplementary Figure S1). Cross-validation was performed with tools from the scikit-learn Python module^1^.

The accuracy was chosen as the characteristic by which the best values of hyperparameters were selected, as calculated by the following formula: Accuracy = (TP + TN)/N, where *N* is the sample size for which the classification was made, and TP and TN are the numbers of correctly defined deleterious mutations and neutral ones, respectively. To select the best classifier, the data for *A. thaliana* were divided into training and validation sets (3409 and 1000 samples, respectively). Classifiers were first trained, and then the classification on the validation set was performed. We used Linear SVM, Gaussian SVM, and RF methods from scikit-learn Python module (see footnote 1); the pipeline for tuning, training and testing the classifiers is available at the GitHub repository https://github.com/kovmax/DelMut.

### Transfer Learning

The transfer learning (TL) is a machine learning technique that improves a model trained on the target data by transfer knowledge from the related and usually larger source data (Pan and Yang, 2010). In our study, we applied TL for training classifiers to predict deleterious mutations in rice and pea datasets (target data) based on the knowledge about deleterious mutations in *A. thaliana* dataset (source data). We examined the Transductive Transfer Learning which assumes that the source data is labeled (classes of samples are known) but the target data is not and, accordingly, labels for the target data were not used until final validation of the predictions. To implement Transductive TL we assign a weight (*W*) for each sample from the source data, which inversely depends on the distance in the feature space from this sample to the mean of the target data domain:

$$\text{W}=\exp(-||x_{i}^{S}-m^{t}|{|}^{2})$$

where $x_{i}^{S}$ is *i*-th sample from the source data, *m*^*t*^ represents mean values of the target dataset features (Pan and Yang, 2010; Lapin et al., 2014). The Transductive TL classifier predicts classes of the target dataset and learns on the weighted source data: the closer a sample form the source data to the target dataset, the more significant it is for training. We applied the Transductive TL technique to Linear SVM, Gaussian SVM, and RF classifiers with hyperparameter values estimated for these classifiers without TL. Methods were implemented with tools of scikit-learn Python module (see footnote 1); all datasets and scripts are available at the GitHub repository https://github.com/kovmax/DelMut.

## Results

### Feature Extraction

**FIGURE 1:**
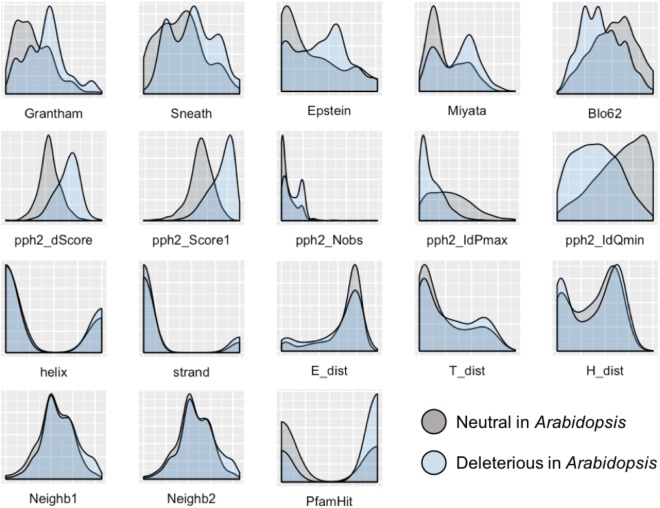
Distribution of features used to characterize the impact of amino acid substitutions in protein sequence for subsets of neutral and deleterious mutations in *Arabidopsis thaliana*. The first row of features – Grantham, Sneath, Epstein, Miyata, and Blo62 (BLOSUM62) – represents distributions of substitution scores based on five corresponding distance matrices. The second row represents the scores obtained with the PolyPhen-2 service: pph2_Score1 and pph2_dScore reflect PSIC scores; pph2_IdPmax, pph2_IdQmin, and pph2_Nobs represent specific features based on the multiple protein alignments. The third row contains features of the secondary protein structure: two features of belonging to helix or strand (helix, strand), and three scores obtained with PCI-SS service (E_dist, T_dist, H_dist). The last row includes two features of the amino acid context around the substitution of interest (Neighb1, Neighb2) and belonging to known Pfam domains (PfamHit). The detailed explanation of features are presented in the Supplementary Table S1.

To develop a method for predicting damaging missense mutations in plants we use machine learning approach and three annotated datasets of non-synonymous deleterious and neutral mutations in *A. thaliana*, *O. sativa*, and *P. sativum* (see Materials and Methods). The method employs classification algorithms and therefore we need to characterize the datasets with a set of features able to discriminate classes. In total, 18 features were selected characterizing the impact of substitution of the wild-type allele by mutant allele on protein sequence and structure. As Figure 1 shows the distributions of all the features differ between subsets of neutral and deleterious mutations in *A. thaliana* that points on their utility for discrimination between these subsets.

### Best Classifier for the *Arabidopsis thaliana* Dataset

The dataset was divided into training and test samples. The test sample was randomly determined, containing 357 neutral and 643 deleterious mutations, and was used to compare the accuracy of the predictions of the four classifiers (PolyPhen-2, Linear SVM, Gaussian SVM, and Random Forest). The results (see Table 1) showed that all the classifiers - Linear SVM, Gaussian SVM, and Random Forest - were more accurate than Polyphen-2, and the most accurate one was Random Forest, it had the highest accuracy and AUC values (ROC-curves are presented in Supplementary Figures S2–S4) and the lowest False Negative and False Positive Rates.

**Table 1:** Performance of four classifiers: PolyPhen2, Linear SVM, Gaussian SVM and Random Forest on the *Arabidopsis thaliana* dataset.

		PolyPhen-2 (PPh2)		Linear SVM (lSVM)		Gaussian SVM (gSVM)		Random Forest (RF)	
		Neutral	Deleterious	Neutral	Deleterious	Neutral	Deleterious	Neutral	Deleterious
Actual classes	**Neutral**	293	164	296	61	301	56	306	3051
	**Deleterious**	1100	543	70	573	74	569	3060	583

Accuracy		0.836		0.869		0.870		300.889	
False Positive Rate (FPR)		0.179		0.171		0.157		300.143	
False Negative Rate (FNR)		0.156		0.109		0.115		300.093	
Sensitivity		0.844		0.891		0.885		300.907	
Specificity		0.821		0.829		0.843		300.857	
AUC		0.907		0.937		0.935		300.952	

### Classification of *Orýza sativa* and *Pisum sativum* With and Without Transfer Learning

**FIGURE 2:**
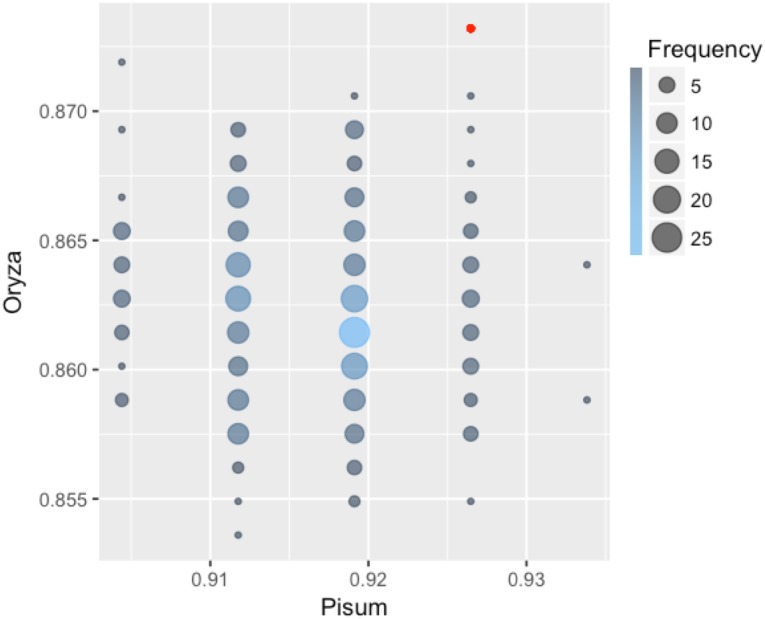
Classification accuracy of 300 Random Forest classifiers learned on the *Arabidopsis thaliana* dataset and applied to classify mutations in pea and rice. Some of the 300 classifiers demonstrated the same values of accuracy on both *Orýza sativa* and *Pisum sativum*. Size and color of circles show frequencies of the classifiers with the same performance. The accuracy value for the best classifier is emphasized with red color.

Each classifier was trained on *Arabidopsis* training samples and applied for prediction in two settings: direct prediction or prediction additionally involving Transfer Learning. Since there is an element of randomization in the Random Forest classification method, estimates for this method were obtained by choosing the best prediction of 300 trained classifiers (Figure 2). By comparing the predicted and annotated class values for the rice and pea mutations, we concluded that the best of the proposed classifiers is Random Forest without the addition of Transfer Learning (Table 2). Predictions of PolyPhen-2 were better only by the criterion False Positive rate, but by the criterion False Negative Rate was significantly underperforming. Overall the Random Forest classifier makes fewer errors in the predictions of a truly deleterious mutation. The prediction of classifiers in the modes without and with Transfer Learning did not exhibit significant differences. Moreover, for the best Random Forest classifier the mode with Transfer Learning turned out to be less accurate.

**Table 2:** Testing classifiers learned on *Arabidopsis* dataset to discriminate deleterious and neutral mutations in rice and pea.

	*Orýza sativa*				*Pisum sativum*			
	Accuracy	FPR	FNR	AUC	Accuracy	FPR	FNR	AUC
PPh2	0.814	300.102	0.270	0.855	0.897	300.044	0.162	0.975

lSVM	0.848	0.144	0.160	0.918	0.912	0.103	0.074	0.971
gSVM	0.842	0.164	0.152	0.890	0.912	0.088	0.088	0.955
RF	300.873	300.115	300.139	300.928	300.926	300.074	300.074	300.981

lSVM + TL	0.848	0.144	0.160	0.918	0.912	0.103	0.074	0.971
gSVM + TL	0.803	0.285	0.110	0.902	0.904	0.147	0.044	0.960
RF + TL	200.861	200.128	200.149	200.926	200.919	200.088	200.074	200.979

*PPh2, PolyPhen-2; lSVM, linear SVM; gSVM, Gaussian SVM; RF, random forest; TL, transfer learning.*

### Classification of Non-synonymous Mutations in *Cicer arietinum*

To test whether or not our classifiers reasonably perform across different angiosperm species, we chose to annotate deleterious mutations in chickpea, *C. arietinum*. Classification has been pursued with both PolyPhen-2 and the Random Forest classifier demonstrated the best discriminating ability on rice and pea datasets (see Figure 2). One may observe (Table 3) that there is a general correspondence between annotations, with 1923 designated as neutral and 851 as deleterious by both classifiers. However, there were also appreciable differences, as may be observed by alternative classifications for 517 mutations. Overall, concordance between two classification results was 84.3%.

**Table 3:** Comparison of the number of deleterious and neutral mutation predicted by PolyPhen-2 and Random Forest classifier in *Cicer arietinum*.

		Random forest	
		Neutral	Deleterious
**PolyPhen-2**	**Neutral**	1923	239
	**Deleterious**	278	851

Due to the lack of annotated missense mutations in chickpea only circumstantial evidence could be used to demonstrate the validity of predictions in this species. To this end, we analyzed the population frequencies of classified polymorphisms in the dataset of 433 chickpea accessions (see Material and Methods). We have calculated the frequencies of synonymous (that are mostly neutral), predicted neutral and predicted deleterious mutations. Due to a large number of missed data, only those genome positions that were called in at least 300 accessions were retained for analysis. Overall, there were 1028 non-synonymous (672 neutral and 356 deleterious) and 901 synonymous polymorphisms (Table 4).

**Table 4:** Mean ffrequencies of non-synonymous deleterious and neutral mutations, as well as synonymous mutations in chickpea dataset.

	Mean frequency
Deleterious	0.050
Neutral	0.097
Synonymous	0.109

Applying the Wilcoxon rank sum test with continuity correction, we showed that there was no statistically significant difference between frequencies of neutral and synonymous substitutions; however, the frequency of deleterious mutations is statistically significantly lower than the frequency of mutations from other classes (one sided test, *P* < 0.05) (Table 5). These results are fully consistent with previous studies on deleterious mutations in other species (Günther and Schmid, 2010; Mezmouk and Ross-Ibarra, 2014) and could be explained by the action of weak purifying selection that sweeps deleterious mutations away. We conclude that our classifier appears to be working across a broad range of angiosperm species.

**Table 5:** Results of the Wilcoxon rank sum test for mutation frequencies comparison.

	Neutral	Synonymous
**Deleterious**	0.036 (<0.05)	0.003 (<0.05)
**Neutral**		0.279 (>0.05)

## Discussion

Here we aimed to develop a classifier specifically tailored for plant datasets that classifies coding non-synonymous mutations into neutral versus functionally deleterious. We have trained the Random Forest classifier in the deleterious mutations in *A. thaliana* using 18 selected features and accomplished a substantially better performance than PolyPhen-2 for two plant species – *O. sativa* and *P. sativum* – for which the sufficient number of neutral and functional mutations is known. The accuracy of our classifier based on Random Forest approach versus PolyPhen-2 was 87% versus 81% for rice and 93% versus 90% for pea. The new classifier also exhibited the superior balance of type I versus type II errors.

We also attempted to improve our classifier using the approaches of Transfer Learning (TL). This has been justified by the following considerations. The task of calling mutation as neutral and deleterious can be set as a classification problem and solved by various methods of machine learning. In mammals, it appeared that the same nucleotide might be deleterious in one species but neutral in another (Kondrashov et al., 2002). Accordingly, training might have to be separately executed species by species. TL appears to be a suitable methodology to implement species-specific training as it could provide knowledge transfer from one species for which à lot of information is available to a close species with limited information. However, here we failed to improve the classifier performance with TL. In fact, the performance of our best Random Forest-based classifier dropped between 1% and 2% for both species, *O. sativa* and *P. sativum*. The reason why TL does not improve classifier performance is not clear. There might be unknown technical reasons, but also some biological considerations. It is known, for instance, that alleles annotated as deleterious in humans at about 15% of cases correspond to normal alleles in other mammals (Kondrashov et al., 2002). Which is to say, as GRNs and proteins diverge between species, the functional importance of different amino acids may also diverge. This might partially be explained by a highly epistatic landscape of amino acid substitutions, as best documented for green fluorescence protein (Sarkisyan et al., 2016). When species with diverged GRNs and proteins mate, their progeny suffer from F1 incompatibility and F2 hybrid breakdown because of epistatic incompatibilities (Turelli and Orr, 2000; Rieseberg and Willis, 2007; Coyne, 2016). It is rather interesting to note that the hybrids between different angiosperm species are much more frequently viable, even at higher phylogenetic distances, than mammals are. In fact, rather than suffering from incompatibilities, plant hybrids may exhibit remarkable hybrid vigor (Garcia et al., 2008; Charlesworth and Willis, 2009) raising a question whether the patterns of GRN and protein divergence in plants are functionally equivalent to those in mammals. It might imply that amino acids substitutions in plant proteins and GRNs are less epistatic, which is to say whether an amino acid substitution is deleterious or not could only weekly change between angiosperm species, unlike mammals. If so, then TL should result in substantial improvements when applied to mammals but not angiosperms. Of course, at this moment, this consideration is nothing more than speculation, but the one deserving attention and specially designed analysis to try the TL methodology in mammals.

While somewhat disappointing, that the classifier works well for different species without the need for species-specific learning also has positive aspects – the classifier does not have to be retrained before applying across angiosperms. To test whether our classifier would work with a new species, we utilized the data on population polymorphisms available for *C. arietinum*. Our hypothesis was that if we annotate these chickpea polymorphisms the population frequency of neutral non-synonymous positions would be identical to the frequencies of synonymous mutations, while the frequencies of functional (i.e., mostly deleterious) mutations would be significantly lower, as these mutations are actively removed by natural selection. This hypothesis was strongly supported, thus the use of our classifier is justified for a broad use with flowering plants.

Overall, our advances open the path to multiple future directions of research. For instance, it would be interesting to infer how different are domesticated plants from their wild progenitors at the genomic level? While it might be assumed that only a few loci contribute to the process of domestication (Gross and Olsen, 2010), domestication can also indirectly affect the entire genome by interfering with natural selection. First, there is strong selection fixing segregating and novel functional alleles. Second, there is an extensive relaxation of natural selection on characters that are important in the wild but not in cultivation, including due to population size reduction. The selective spread of beneficial mutations but also a consequent build-up of deleterious mutations (especially closely linked to selective sweeps) have been well-documented in plants, including rice (Günther and Schmid, 2010) and maize (Pyhäjärvi et al., 2013). However, whether deleterious mutation build-up is a minor nuisance or a major drag on yield remains incompletely understood, and can now be researched. This will help to understand whether ‘cleaning out’ such adverse mutations, for instance with CRISPR-based tools, might contribute to substantial gains in yield. Further, it opens the way to prioritizing these mutations for being edited out – perhaps of substantial value to the workflow in future agricultural advances.

## Author Contributions

MK and AI have contributed equally to this work. MS and SN supervised the study.

## Conflict of Interest Statement

The authors declare that the research was conducted in the absence of any commercial or financial relationships that could be construed as a potential conflict of interest.

## Footnotes

1 http://scikit-learn.org
