# Supplementary material for: A Pipeline for Classifying Deleterious Coding Mutations in Agricultural Plants
Source: Front Plant Sci. 2018 Nov 28;9:1734. doi: 10.3389/fpls.2018.01734 (PMC6279870; doi:10.3389/fpls.2018.01734)
Supplement: Supplementary file 1 [file Data_Sheet_1.PDF]

Supplementary Tables

Table S1. Description of classification features

	Name of the feature	Description	Reference
1	Grantham	Grantham score of substitutions	(Grantham, 1974)
2	Sneath	Sneath score of substitutions	(Sneath, 1966)
3	Epstein	Epstein score of substitutions	(EPSTEIN, 1967)
4	Miyata	Miyata score of substitutions	(Miyata et al., 1979)
5	Blo62	BLOSUM62 score of substitutions	(Henikoff and Henikoff, 1992)
6	pph2_Score1	PSIC score for wild type amino acid residues	(Adzhubei et al., 2010)
7	pph2_dScore	difference of PSIC scores for two amino acid residue variants	
8	pph2_IdPmax	Maximum congruency of the mutant amino acid residue to all sequences in multiple alignment	
9	pph2_IdQmin	Query sequence identity with the closest homologue deviating from the wild type amino acid residue	
10	pph2_Nobs	Number of residues observed at the substitution position in multiple alignment (without gaps)	
11	helix	Binary variable: whether or not a substitution locates within the helix of the secondary structure predicted by PfamScan	(Finn et al., 2014)
12	strand	Binary variable: whether or not a substitution locates within a strand of the secondary structure predicted by PfamScan	
13	E_dist	PCI-SS score for β-strands	(Green et al., 2009)
14	T_dist	PCI-SS score for non-regular structures	
15	H_dist	PCI-SS score for α-helices	
16	Neighb1	Mean Grantham score between the wild type amino acid residue and two neighbor amino acid residues	New features
17	Neighb2	Mean Grantham score between the wild type amino acid residue and two amino acid residues separated from the target by one amino acid position	
18	PfamHit	Belonging to known Pfam domains	(Finn et al., 2014)

Supplementary Figures

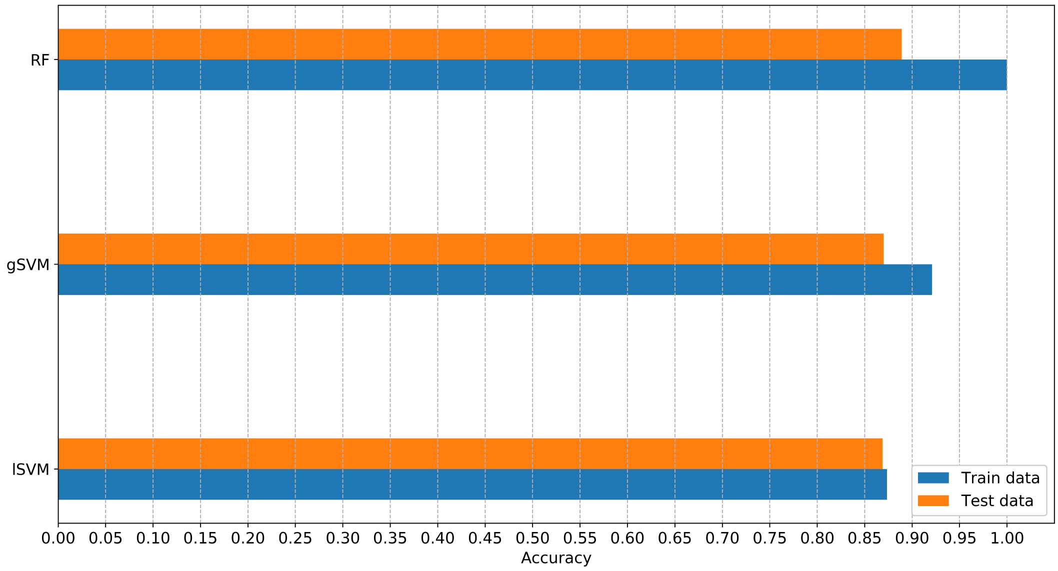

Figure S1. Accuracy values obtained after training and testing different classifiers on the *A. thaliana* dataset: Linear SVM (**lSVM**), Gaussian SVM (**gSVM**) and Random Forest (**RF**). Due to the comparable accuracy values calculated for train and test sets using the optimal values of hyperparameters, we concluded, that the models avoided the overfitting.

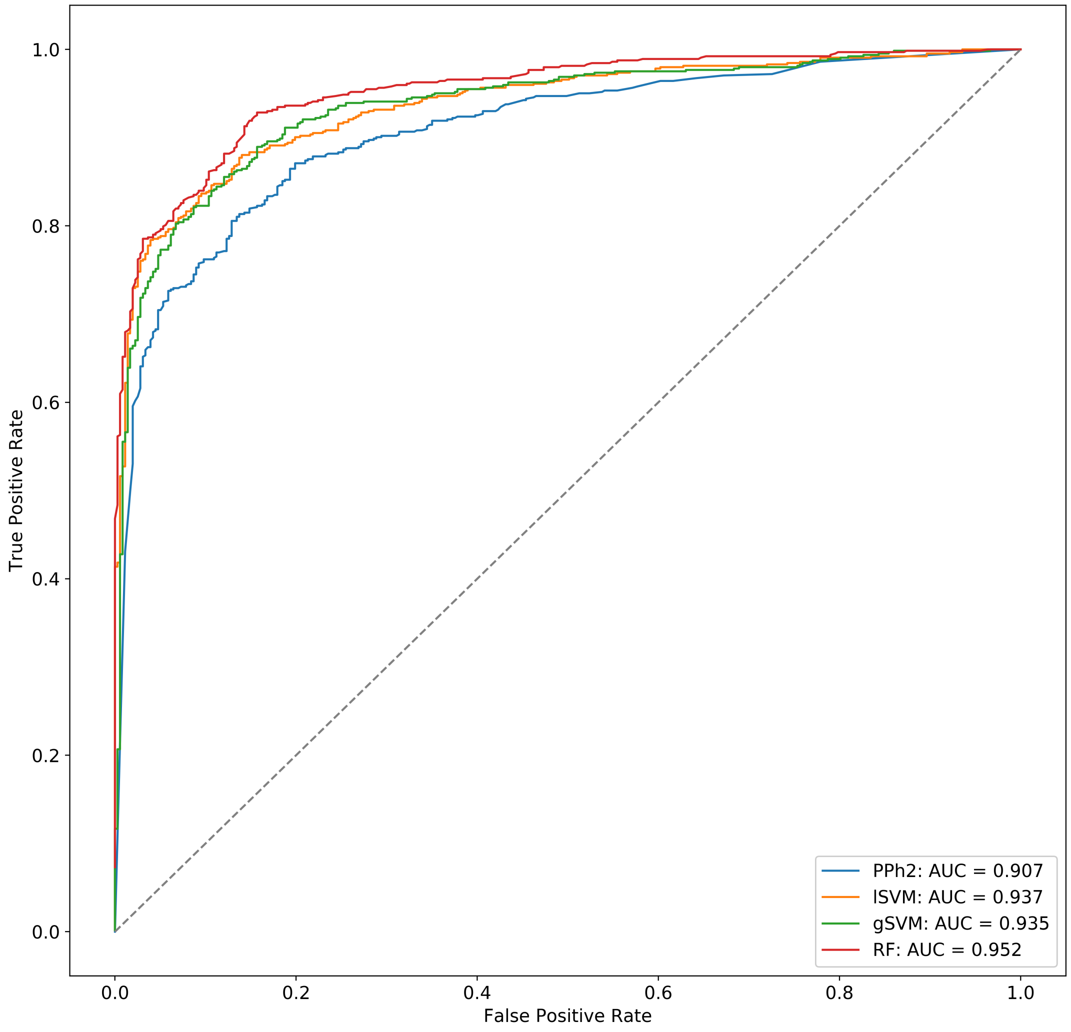

Figure S2. ROC-curves for PolyPhen-2 (**PPh2**), Linear SVM (**lSVM**), Gaussian SVM (**gSVM**) and Random Forest (RF), which were used for the mutation prediction of *A. thaliana* test dataset. The dashed line accounts for the ROC-curve for a random guessing. The legend contains Area Under Curve (**AUC**) values corresponding to the each classifier in question.

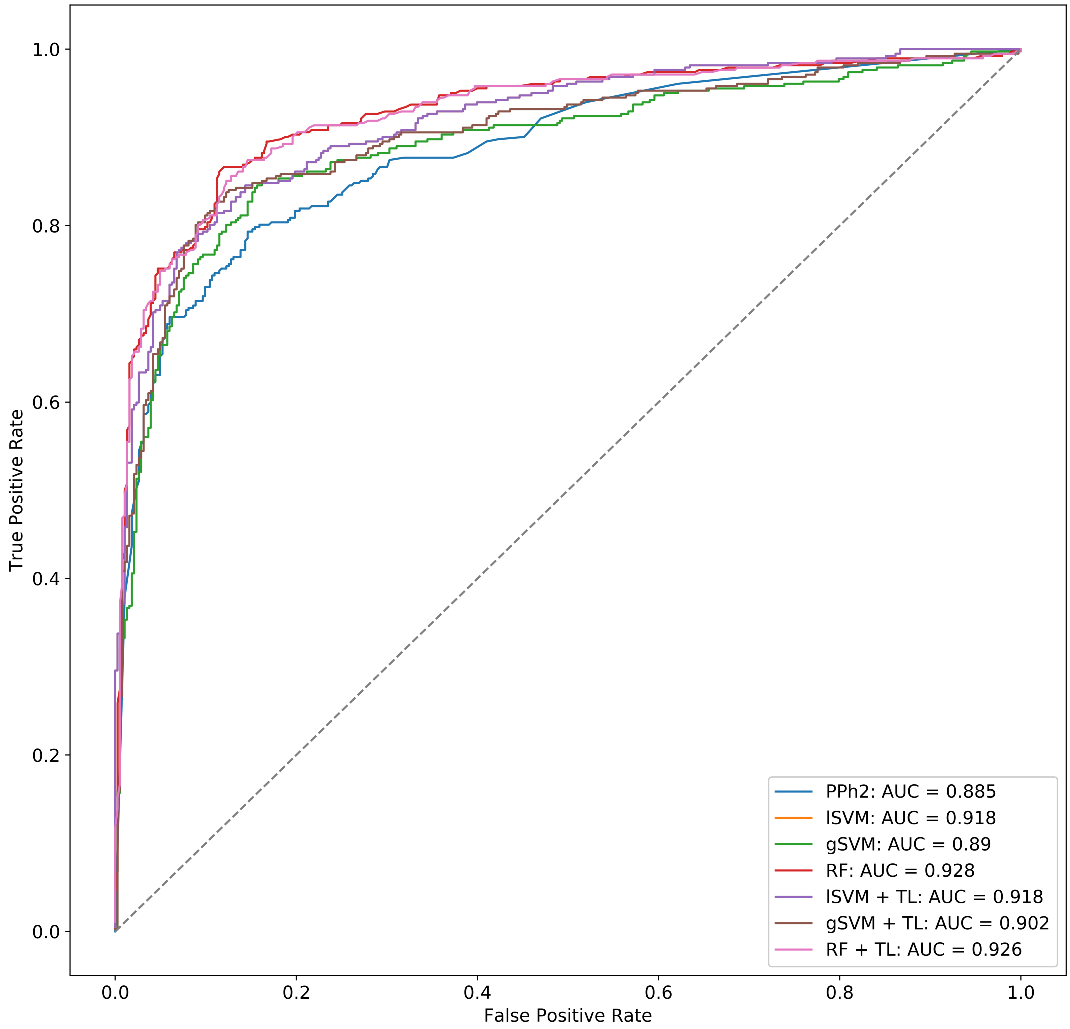

Figure S3. ROC-curves for PolyPhen-2 (**PPh2**), Linear SVM (**lSVM**), Gaussian SVM (**gSVM**), Random Forest (**RF**) and the last three classifiers with applying Transfer Learning (**lSVM + TL**, **gSVM + TL**, **RF + TL**, respectively), which were used for the mutation prediction of *O. sativa* data. The dashed line accounts for the ROC-curve for a random guessing. The legend contains Area Under Curve (**AUC**) values corresponding to the each classifier in question.

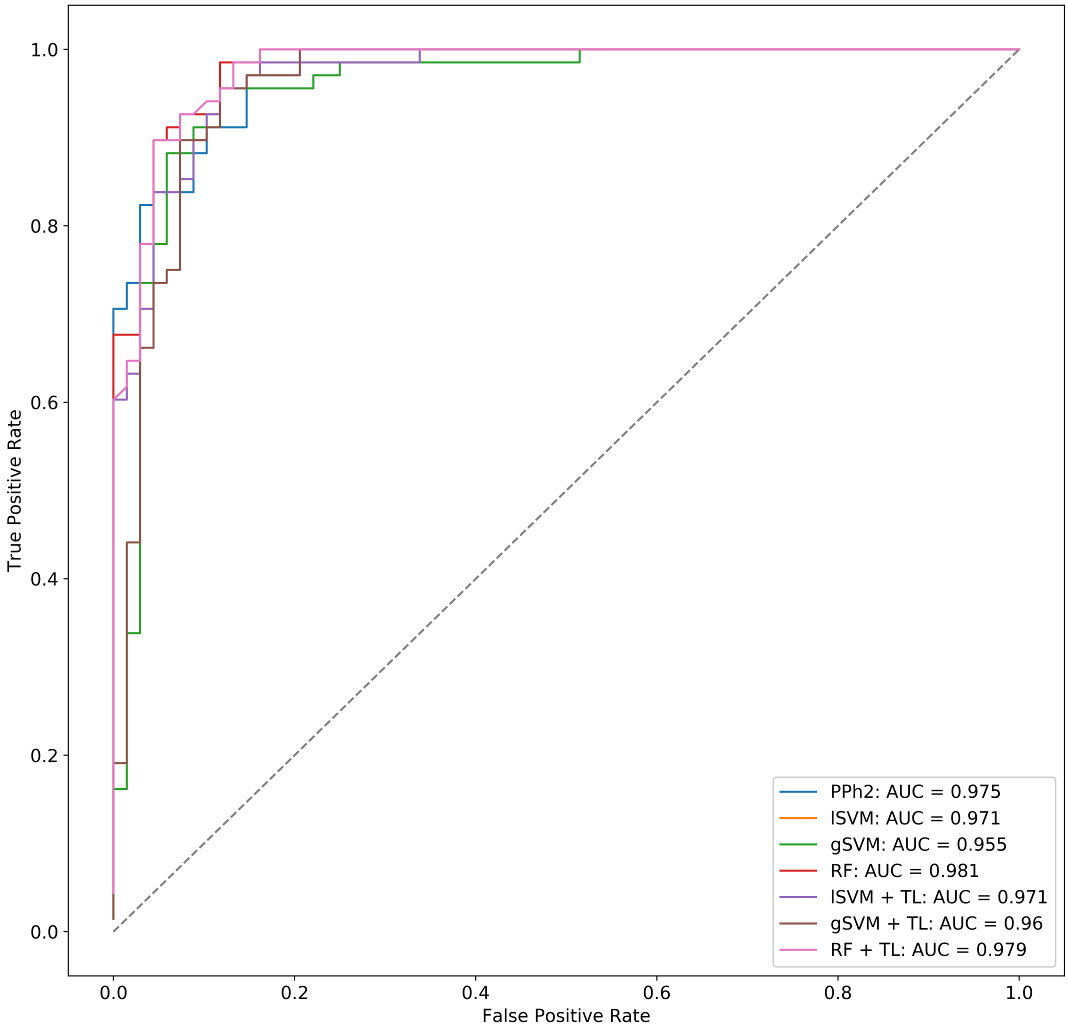

Figure S4. ROC-curves for PolyPhen-2 (**PPh2**), Linear SVM (**lSVM**), Gaussian SVM (**gSVM**), Random Forest (**RF**) and the last three classifiers with applying Transfer Learning (**lSVM + TL**, **gSVM + TL**, **RF + TL**, respectively), which were used for the mutation prediction of *P. sativum* data. The dashed line accounts for the ROC-curve for a random guessing. The legend contains Area Under Curve (**AUC**) values corresponding to the each classifier in question.

References

Adzhubei, I. A., Schmidt, S., Peshkin, L., Ramensky, V. E., Gerasimova, A., Bork, P., et al. (2010). A method and server for predicting damaging missense mutations. *Nat. Methods* 7, 248–249. doi:10.1038/nmeth0410-248.

EPSTEIN, C. J. (1967). Non-randomness of Ammo-acid Changes in the Evolution of Homologous Proteins. *Nature* 215, 355–359. doi:10.1038/215355a0.

Finn, R. D., Bateman, A., Clements, J., Coggill, P., Eberhardt, R. Y., Eddy, S. R., et al. (2014). Pfam: the protein families database. *Nucleic Acids Res.* 42, D222–D230. doi:10.1093/nar/gkt1223.

Grantham, R. (1974). Amino acid difference formula to help explain protein evolution. *Science* 185, 862–4.

Green, J. R., Korenberg, M. J., and Aboul-Magd, M. O. (2009). PCI-SS: MISO dynamic nonlinear protein secondary structure prediction. *BMC Bioinformatics* 10, 222. doi:10.1186/1471-2105-10-222.

Henikoff, S., and Henikoff, J. G. (1992). Amino acid substitution matrices from protein blocks. *Proc. Natl. Acad. Sci.* 89, 10915–10919. doi:10.1073/pnas.89.22.10915.

Miyata, T., Miyazawa, S., and Yasunaga, T. (1979). Two types of amino acid substitutions in protein evolution. *J. Mol. Evol.* 12, 219–236. doi:10.1007/BF01732340.

Sneath, P. H. (1966). Relations between chemical structure and biological activity in peptides. *J. Theor. Biol.* 12, 157–95. doi:4291386.
